# Novel hemostatic patch achieves sutureless epicardial wound closure during complex cardiac surgery, a case report

**DOI:** 10.1186/s13019-015-0215-z

**Published:** 2015-01-27

**Authors:** Jayant S Jainandunsing, Sali Al-Ansari, Bozena D Woltersom, Thomas WL Scheeren, Ehsan Natour

**Affiliations:** 1 Department of Anesthesia and Pain Medicine, University Medical Centre Groningen, Groningen, The Netherlands; 2 Department of Cardio-Thoracic Surgery, University of Groningen, Groningen, The Netherlands

**Keywords:** Hemostasis, Epicardial wound closure, Sutureless repair

## Abstract

Treatment of damaged cardiac tissue in patients with high bleeding tendency can be very challenging, damaged myocardial tissue has a high rupture risk when being sutured subsequently on-going bleeding is a major risk factor for poor clinical outcome. We present a case demonstrating the feasibility in using a novel haemostatic collagen sponge for the management of a myocardial wound. This report is the first description in cardiac surgery where Hemopatch® sponges are used to successfully seal a left ventricle wound. Our patient was diagnosed with endocarditis, had a low pre-operative haemoglobin count and underwent cardiac surgery for multiple valve repairs. The procedure was performed on cardiopulmonary bypass, which meant our patient had to be heparinized. Despite these major risk factors for bleeding Hemopatch® managed to contain bleeding and seal the wound, no sutures were needed.

## Background

Management of patients on cardiopulmonary bypass (CPB) can be very challenging with regards to haemostasis. Especially endocarditis patients with long CPB time while being heparinised and cooled. Although surgeons are aware of certain sites prone to bleeding, such as cannulation sites, suture areas and bone, vigilance should be maintained at all times for other less susceptible areas. These areas could include anatomical sites where optimal suture techniques cannot be applied without risking damages to newly positioned mechanical valves or annular rings. Often a compromise is sought trying to lift the heart for optimal haemostasis while minimizing cardiac manipulation. An alternative technique that has become feasible now, is a sutureless approach using novel patches, with good adhesive and haemostatic properties. Allowing surgeons to reach difficult suturing areas without compromising cardiac function.

## Case presentation

A 64-year-old man with signs of severe weight loss, dyspnoea, cough and edema of the lower extremities was referred to our institute. Laboratory analysis revealed leucocytosis (12 · 7 × 10^9^/l), raised C-reactive protein levels (64 mg/l), and decreased hemoglobin concentration (6 · 0 mmol/l). His medical history included hypertension, systemic vascular disease and hereditary hypertrophic obstructive cardiomyopathy.

Echocardiography indicated severe mitral and aortic insufficiencies secondary to partial mitral leaflet and aortic cusp destruction. Systolic anterior motion of the anterior mitral leaflet was also observed, with subsequent partial obstruction of the left ventricular outflow tract. Coronary angiography demonstrated significant left anterior descending (LAD) and anterolateral (AL) coronary artery lesions. Blood cultures were positive for Streptococcus gordonii.

We planned to graft the LAD and AL coronary arteries, partially resect the upper part of the inter-ventricular septum, following the Morrow procedure and replace both the aortic and mitral valve.

**Figure 1:**
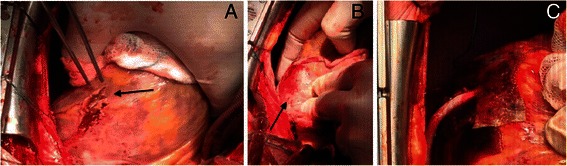
**Sutureless epicardial wound closure with Hemopatch. A**. Epicardial exposure of the anterolateral coronary artery (arrow). **B**. Wound area is covered with Hemopatch just beneath the venous anastomosis (arrow). **C**. A second Hemopatch is positioned around the venous anastomosis, with hemostasis achieved while the coronary flow is established. Full flow can be seen in the venous anastomosis (compare with B, where it is flaccid).

Endocarditis was suspected during surgery and a high bleeding tendency was observed. A bleeding occurred originating from an intramuscular anterolateral coronary artery, during exposure of the vessel and secondary to friable myocardial tissue (Figure 1A). This specific area became prone to diffuse bleeding. It was a combined arterial-venous bleeding. Decision was made to use the resorbable, collagen-based Hemopatch® Sealing Hemostat (Baxter International, Deerfield, IL, USA,) to achieve haemostasis without suturing.

After the AL venous anastomosis, while the patient was heparinized and on cardiopulmonary bypass (CPB), two patches were used (Figure 1B,C): one directly over the dissected area; and the second around the anastomosis after making a Y-shaped cut in the patch. The patch was placed dry and centred on the bleeding focus, after positioning we immediately applied pressure locally, using dry gauze for 2 minutes. Care was taken to avoid disruption of the locally formed clot.

Activated clotting time levels during perfusion were around 500 seconds, and 130 seconds at the end of surgery. Post-operative coagulation parameters were PT 12.1 seconds, aPTT 56 seconds, Fibrinogen 2.7, these values were corrected towards normal values within 6 hrs.

Our patient received a total of 6 units of fresh frozen plasma (300 ml each), 5 units of red blood cells (275 ml each), one unit of Thrombocytes (310 ml), 2 times 2gr tranexamic acid, and 2 grams of fibrinogen. All these products were given during surgery and within the first 6 hours on the ICU.

Fourteen hours post-surgery patient was transferred to the ward without any bleeding or other complications. Six days post surgery a routine transthoracic echocardiographic examination was performed, no pericardial effusion was seen and neither were any pseudo aneurysm formation detected, patient was discharged home with no valve anomalies, with a sinus rhythm and in good clinical condition.

## Conclusions

CPB surgery carries a high post-surgical bleeding risk that is exacerbated by long lasting procedures [1,2]. Our case was a challenge for the whole surgical team. Multiple cardiac corrections resulted in 5 hours lasting CPB. Bleeding risk was also increased by patient’s inflammatory state, which is associated with coagulation system activation and clotting factor depletion and a low haemoglobin count, a known risk factor for haemorrhage [2-4]. Another issue was that all patients are heparinised while on CPB, and after CPB is terminated, heparin is inactivated using protamine. However protamine’s short half-life can cause a re-activation of heparin increasing the risk of post-surgical bleeding [5].

Our treatment options were, suturing the wound, using glue, patches or assess the effect of protamine. Suturing was not an option due to friable tissue and risk of graft dysfunction In our experience fibrin glue was not indicated in this case because bleeding could wash out the fibrin. We wanted to be certain that the bleeding was effectively treated, preference was given to a patch above glue.

Protamine can be effective in many cases, but in our case it would mean taking a risk, because if protamine would not work re-luxation of the heart would be necessary increasing the risk of ventricle wall damage due to the prior implanted mitral valve prosthesis.

Performing mitral valve replacements made treating the lateral cardiac wall more challenging. Lateral wall exposure requires lifting the heart, thus increasing the risk of mitral valve disruption and subsequent ventricular damage. We therefore concluded that a patch would be safer option than attempting suturing, in preserving valve integrity. The reason we choose for Hemopatch® was because it is a patch that can be used dry, allowing it to be cut and remodelled easily, thus providing better handling opportunity compared to patches that have to be applied wet.

The patch achieved local haemostasis during surgery and subsequently maintained wound closure. Blood loss on the intensive care unit was minimal (260 ml during the first 6 hours). Crucially, the patch around the anastomosis did not interrupt coronary blood flow within the graft.

This case is the first in which Hemopatch has been used in humans to achieve successful sutureless hemostasis during cardiac surgery. Although this type of complex case is not one every surgeon comes across regularly, it highlights how a simple and adaptable patch can be beneficial in patients with a high bleeding risk. This case is another sample of how collagen sponges can achieve sutureless haemostasis. Unlike other existing patches, which are used off-label, to achieve sutureless haemostasis, hemopatch is specifically designed for a sutureless approach, not only in cardiac surgery but also for other surgical specialties.

## Consent

Written informed consent was obtained from all patients for publication of this case report and any accompanying images. A copy of the written consent is available for review by the Editor-in-Chief of the journal of cardiothoracic surgery.

## Ethical committee

No approval was required from our ethical committee, since this case wasn’t part of any clinical trial.

## Abbreviations

CPB: Cardio-pulmonary bypass
LAD: Left anterior descending coronary artery
AL: Antero-lateral coronary artery
PT: Prothrombine time
aPTT: Activated partial thromboplastine time
